# Predicting the risk of sarcopenia in elderly patients with patellar fracture: development and assessment of a new predictive nomogram

**DOI:** 10.7717/peerj.8793

**Published:** 2020-04-15

**Authors:** Yi-sheng Chen, Yan-xian Cai, Xue-ran Kang, Zi-hui Zhou, Xin Qi, Chen-ting Ying, Yun-peng Zhang, Jie Tao

**Affiliations:** 1 Department of Orthopedics, Shanghai General Hospital, Shanghai Jiao Tong University School of Medicine, Shanghai, China; 2 Department of Plastic Surgery, Shanghai General Hospital, Shanghai Jiao Tong University School of Medicine, Shanghai, China; 3 Department of Otolaryngology-Head and Neck Surgery, Shanghai Ninth People’s Hospital, Shanghai Jiao Tong University School of Medicine, Shanghai, China; 4 Ear Institute, Shanghai JiaoTong University School of Medicine, Shanghai, China; 5 Shanghai Key Laboratory of Translational Medicine on Ear and Nose diseases, Shanghai, China

**Keywords:** Patellar fracture, Sarcopenia, Nomogram prediction model, Skeletal muscle, Logistic regression model

## Abstract

**Purpose:**

To develop a risk prediction model for postoperative sarcopenia in elderly patients with patellar fractures in China.

**Patients and methods:**

We conducted a community survey of patients aged ≥55 years who underwent surgery for patellar fractures between January 2013 and October 2018, through telephone interviews, community visits, and outpatient follow-up. We established a predictive model for assessing the risk of sarcopenia after patellar fractures. We developed the prediction model by combining multivariate logistic regression analysis with the least absolute shrinkage model and selection operator regression (lasso analysis) as well as the Support Vector Machine (SVM) algorithm. The predictive quality and clinical utility of the predictive model were determined using C-index, calibration plots, and decision curve analysis. We also conducted internal sampling methods for qualitative assessment.

**Result:**

We recruited 137 participants (53 male; mean age, 65.7 years). Various risk factors were assessed, and low body mass index and advanced age were identified as the most important risk factor (*P* < 0.05). The prediction rate of the model was good (C-index: 0.88; 95% CI [0.80552–0.95448]), with a satisfactory correction effect. The C index is 0.97 in the validation queue and 0.894 in the entire cohort. Decision curve analysis suggested good clinical practicability.

**Conclusion:**

Our prediction model shows promise as a cost-effective tool for predicting the risk of postoperative sarcopenia in elderly patients based on the following: advanced age, low body mass index, diabetes, less outdoor exercise, no postoperative rehabilitation, different surgical methods, diabetes, open fracture, and removal of internal fixation.

## Introduction

In 2010, the European Working Group on Sarcopenia in Older People (EWGSOP) and the International Symposium on Sarcopenia in 2011 established a unified definition of sarcopenia, which highlighted that sarcopenia occurs as a result of a gradual decline in muscle mass. At the beginning of 2018, the Working Group met again (EWGSOP2) to summarize the latest research results of clinical and scientific research on sarcopenia in the past 10 years and revise the diagnostic criteria (Cruz-Jentoft et al., 2019). Sarcopenia is characterized by a gradual and generalized loss of the strength and function of skeletal muscles (Bianchi et al., 2016; Cruz-Jentoft et al., 2010). It has be closely associated with diabetes as well as the risk of subsequent falls, fractures, and physical disabilities (Cruz-Jentoft et al., 2010; Daly et al., 2014; Janssen et al., 2004; Kanazawa et al., 2008; Molina et al., 2017). Therefore, the prevention of sarcopenia can help reduce the risk of falls and secondary fractures, thereby improving the overall prognosis of patients with fractures (McKinnon et al., 2017; Paillard, 2017).

The occurrence of fractures is an important risk factor for sarcopenia (Szulc, Feyt & Chapurlat, 2016). Patellar fracture is a common fracture of the lower limb (Luna-Pizarro et al., 2006) and may entail long-term bed rest for patients, which is an important cause of sarcopenia (Changjie, 2014). The patella is an important component of the knee joint, playing an important role in the attachment of the quadriceps muscle to the patellar ligament and thereby maintaining the stability and functioning of the knee joint. About 40% of the patients with patella fracture have a decrease in knee strength and endurance in the first year (Rafael & Archdeacon, 2016). Thus, patients with patella fracture may be prone to sarcopenia. Therefore, we believe that studying patella fracture may be more useful than other types of limb fractures to identify the risk factors of sarcopenia after lower limb fracture.

However, the pathogenesis of sarcopenia is multi-centric involving various factors, including those related to disease (such as health status and work intensity), treatment (such as surgery and rehabilitation programs and other medical-related issues), and patients (such as age, body mass index (BMI), gender, and education level). In addition, ageing and diabetes have also been implicated as important risk factors for sarcopenia (Barzilay et al., 2009; Kalyani, Corriere & Ferrucci, 2014; Kuo et al., 2009). The risk of sarcopenia is reported to be high among patients with diabetes (Kim et al., 2010). Decrease in muscle mass and the resulting abnormal gait and slow walking speed has been reported as the mechanism underlying sarcopenia (Volpato et al., 2012).

Although several variables that affect sarcopenia have been reported, no method has yet been identified for systematic assessment to predict the risk of post-fracture muscle reduction; therefore, it is necessary to develop accurate predictive tools and early individualized interventions to prevent muscle loss. The Asian Working Group for Sarcopenia (AWGS) and EWGSOP2 established effective criteria for the diagnosis of sarcopenia based on the characteristics of the Asian population; these criteria are being widely used to assess the occurrence and risk factors of sarcopenia (Chen et al., 2014). We believe that the AWGS criteria can be used to predict the occurrence of sarcopenia after patellar surgery by establishing an effective nomogram model.

The aim of this study was to develop an effective and simple tool for predicting sarcopenia in patients undergoing post-fracture patellar surgery. Only the data available for period after the patient’s hospitalization for fracture were used to evaluate the risk of sarcopenia in patients with patella fracture in order to study the effects of early prevention of sarcopenia and obtain accurate guidance for clinical work.

## Patients and Methods

### Patients

This study was designed as a retrospective investigation of the prognosis of elderly patients with patella fracture. The study protocol was approved by the ethics committee of the First People’s Hospital affiliated to Shanghai Jiao Tong University Medical School (approval no. 2019SQ059). The study was performed in accordance with the stipulations of the Helsinki declaration. We first identified patients residing in Shanghai, China, who underwent patellar surgery at the Shanghai First People’s Hospital between January 2013 and October 2018. Between February 2019 and June 2019, we conducted a questionnaire-based survey through appointment-based telephonic interview, outpatient services, and community follow-up. Data for all the patients were collected by the same researcher. Written informed consent was sought from all enrolled subjects before participation in the study; all participating patients provided consent. In this study, we included only fracture patients aged ≥55 years, since postoperative sarcopenia after fractures is rare in young patients (Alkahtani, 2017; Frost et al., 2015; Krzyminska-Siemaszko et al., 2019). The laboratory blood test results taken into consideration were those obtained for the preoperative fasting samples of venous blood.

### Inclusion criteria and exclusion criteria

The inclusion criteria for the survey were as follows: (1) diagnosis of sarcopenia according to the AWGS criteria, (2) age ≥55 years, (3) availability of complete data for baseline clinical characteristics (age, body mass index, etc.) and follow-ups, (4) patients had been managed with self-care before surgery, (5) basic communication skills.

Patients were excluded from the study if they had major illnesses, serum creatinine levels of >2.5 mg/dL, liver enzyme levels elevated to more than twice the upper limit of the normal within 6 months prior to the start of the study, unexplained fever or infection, acute heart failure, or organ failure such as renal failure. Patients were also excluded if they did not cooperate with the completion of the basic diagnostic tests. Patients who developed life-threatening conditions during follow-up or conditions that could interfere with the interpretation of the study results were also excluded.

All patients were screened, of which 137 were found to be eligible according to the inclusion criteria and availability of the completed questionnaire. The patients included in the final analysis included 52 males (age: 55–88 years, mean age: 66.1 ± 9.2 years) and 85 females (age: 55–88 years, average age: 65.4 ± 8.1 years). These 137 patients were included in the final analysis.

### Methods of assessment

The diagnostic criteria established by the 2014 Asian Working Group for Sarcopenia (AWGS) and EWGSOP2 defines sarcopenia by a walking speed of ≤0.8 m/s and measuring muscle mass (BIA) of ≤7.0 kg/m^2^ for males and ≤6.0 kg/m^2^ for females (Cruz-Jentoft et al., 2019; Chen et al., 2014). The pace of decline in walking speed is closely related to the prognosis of patients (Stone et al., 2015; Kutner et al., 2014). Hence, we used a fixed distance of 6 m, as recommended by AWGS, to measure the subject’s daily walking speed. For patients with a walking speed of ≤0.8 m/s, we used the bioelectrical impedance analysis (BIA) to assess muscle mass. The results of BIA are very similar to those of double-energy X-ray absorptiometry and magnetic resonance imaging; BIA also offers the advantages of safety, technical simplicity, low cost, and high patient compliance (Wang et al., 2016; Chien, Huang & Wu, 2008). All the results of BIA have been standardized by using cross-validated Sergi. Muscle mass and BMI of the extremities were measured by using a bioimpedance meter (TANITA RD-953, Japan).

### Medical history and basic data collection

The questionnaire contained questions pertaining to various domains, including history of hypertension, diabetes, osteoporosis, malignant tumors, myocardial infarction, and falls as well as educational level, income, and duration of exercise. With regard to smoking habit, patients were classified as current smokers and non-smokers. Alcohol intake was calculated according to the average weekly alcohol consumption. The cut-off for exercise duration was 2 h/week, with duration of <2 h/week being considered as reduced duration. All patients had medical insurance that is provided for Shanghai residents. Additionally, we also reviewed the patients’ medical history and collected their laboratory blood test results.

### Statistical analysis

The study cohort was randomized into the training and validation (7:3) groups, which were used for diagnostic and prognostic analysis and to identify and evaluate the model. Statistical analysis was performed using R software (version 3.5.3). All the pre-determined factors described above were included in the LASSO analysis and to reduce the dimensionality of the data, and the best predictors were identified (odds ratio of the OR values of these characteristic factors have 95% confidence intervals and *P* values <0.05) (Friedman, Hastie & Tibshirani, 2010; Kidd et al., 2018; Sauerbrei, Royston & Binder, 2007). These features were then further filtered using the Support Vector Machine-Recursive Feature Elimination (SVM-RFE) algorithm. Subsequently, the multivariate logistic model was used to analyze the factors identified with the LASSO regression model and Support Vector Machine (SVM) algorithm to establish a prediction model (Kidd et al., 2018). The collected patient data can be used to generate a predictive model that accounts for all the best predictors of sarcopenia, which can be used for the prognostic evaluation of patients with patellar fractures (Iasonos et al., 2008; Balachandran et al., 2015). Next, we plotted the calibration curve to assess the accuracy of the nomogram. However, simple evaluation with the calibration curve would be incomplete (Kramer & Zimmerman, 2007). In addition, we also calculated the C-index and plotted the area under curve (AUC) curve to quantify the predictive power of the nomogram. We then performed further iterations (10,000 resampling) on the nomogram using the R language package for a more accurate measurement of C-index (Pencina & D’Agostino, 2004). Finally, we used the decision curve model to determine the net rate of return in order to test the clinical utility of the nomogram (Vickers et al., 2008). By quantifying the net benefit of different threshold probabilities in patient information, decision curve analysis is used to assess the clinical utility of nomograms (Huang et al., 2016; Van Calster et al., 2018).

## Results

### Basic characteristics of the data

We obtained the completed questionnaires from 137 patients (52 males, 85 females; mean age 65.7 years) with patellar fractures. Patients were classified as those with (45 patients) or without sarcopenia (92 patients), as according to the diagnostic criteria established by the AWGS and EWGSOP2. Details of the patient characteristics are provided in Table 1.

### Screening of predictors

**Figure 1:**
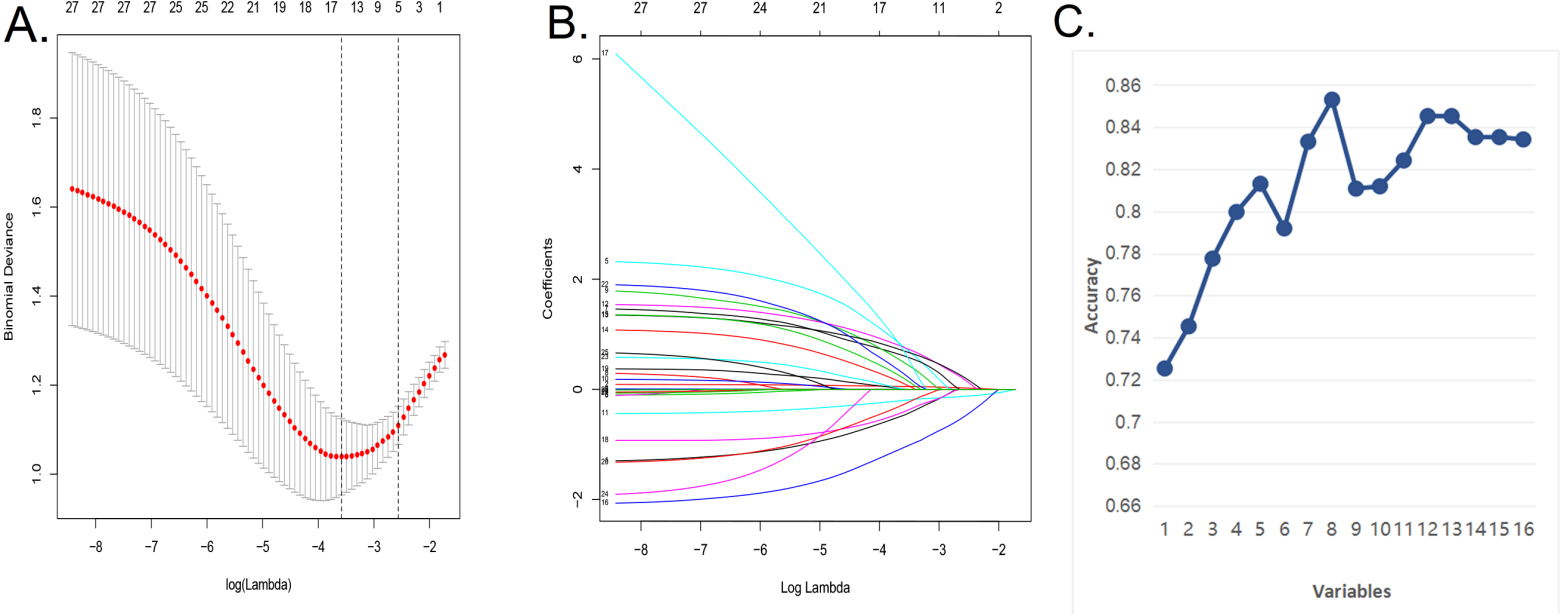
Demographic and clinical feature selection using the LASSO binary logistic regression model and support vector machine algorithm. (A) In the Lasso model, the choice of the optimal parameters used a five-fold cross-validation approach. Using the partial likelihood anomaly curve and the log (lambda) plot, the vertical line is drawn at the optimal value to obtain the included feature factors. (B) The lambda curve generates a profile based on the log (lambda) sequence. Vertical lines were drawn at the values selected by the five-fold cross-validation method, and 16 characteristic factors were selected. (C) Using the support vector machine SVM-RFE algorithm to further screen these 16 characteristic factors, we finally established a prediction model with eight best features with an average 10-fold cross-validation score of 0.8531.

We used the LASSO regression model and SVM algorithm (Figs. 1A–1C) to decrease the factors in this study from 28 to 8 major parameters, which were age, BMI, diabetes, postoperative rehabilitation, frequency of outdoor exercise, surgical method used, presence or absence of open fracture, and removal of internal fixation.

### Construction of a personalized prediction model

**Figure 2:**
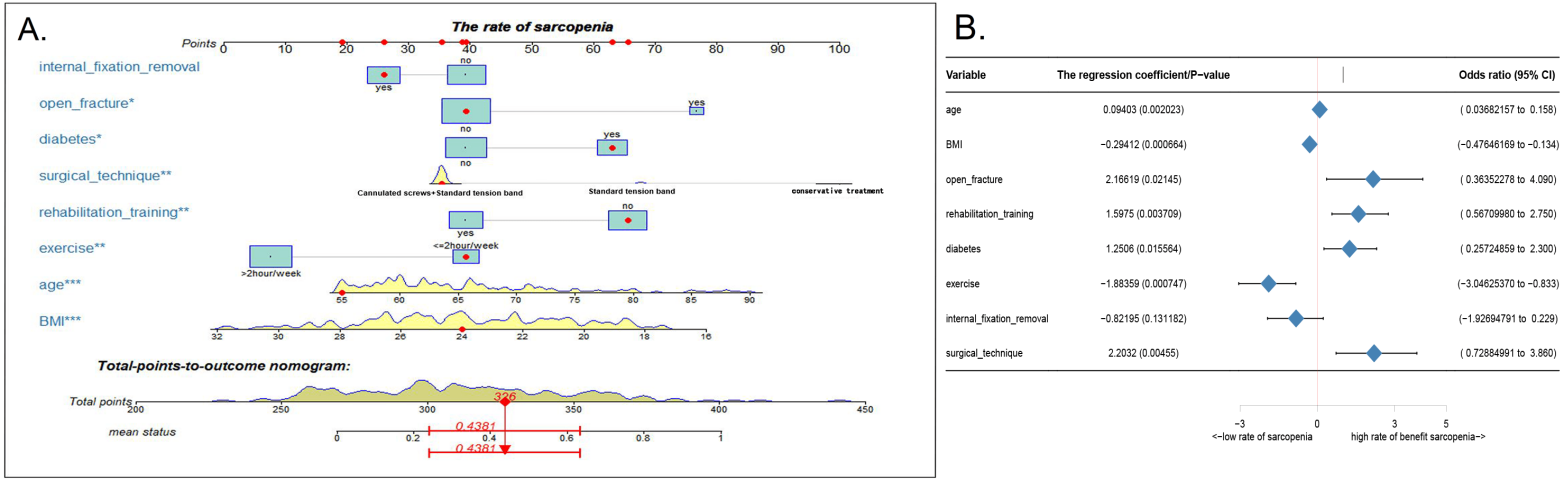
Nomogram model and prediction factors for sarcopenia in elderly patients with patellar fracture. (A) Nomogram model to predict the risk of sarcopenia after patella fracture. Note: eight factors including age, BMI, diabetes, postoperative rehabilitation, frequency of outdoor exercise, surgical method used, presence or absence of open fracture, and removal of internal fixation were included. (B) The forest chart of prediction factors for sarcopenia in elderly patients with patellar fracture.

Using the eight abovementioned predictors identified with the logistic model analysis, we used the RMS package of R software to construct the column diagram, as shown in Figs. 2A (see 2B for details).

### Evaluation the nomogram

**Figure 3:**
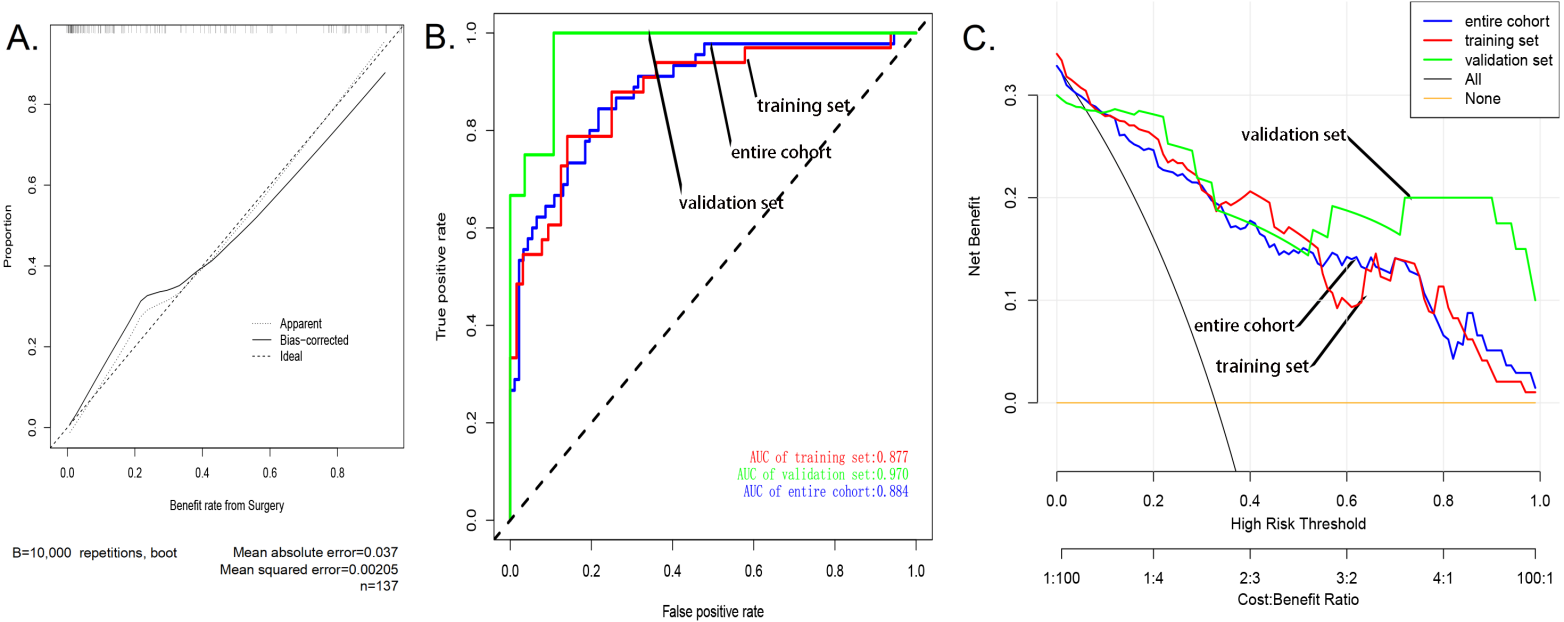
Evaluation of the nomogram prediction model. (A) A calibration curve for the predictive model of sarcopenia in cases of patella fracture. Note: The *x*-axis is the risk of sarcopenia. The *y*-axis represents the actual incidence of sarcopenia. The diagonal dashed line represents a perfect prediction of an ideal model. The solid line indicates the predictive power of this predictive model, and the more it fits with the dotted line, the better the predictive ability. (B) The area under the curve (AUC) of the sarcopenia prediction model indicates the probability of accurately predicting whether or not the patient has sarcopenia in a randomly selected case. The model exhibited good predictive power, and the AUC values of the training group (red), the test group (green), and the whole sample (blue) are 0.8917397, 0.9476584, and 0.9114173, respectively. (C) The decision curve of the nomogram. The figure shows the decision analysis curves for training, test, and overall groups.

The calibration curve of the nomogram showed good agreement (Fig. 3A), which suggested that this model could be used to predict the risk of sarcopenia in patients after patellar fracture. In addition, the AUC of this prediction model was 0.884058 (receiver operating curve shown in Fig. 3B). Further, the model showed a good prediction rate (C-index: 0.88; 95% CI [0.80552–0.95448]) and good correction effect. The C index was 0.97 for the validation queue and 0.894 for the entire cohort. This proved that this model was very efficient in terms of predictive power.

### Clinical application

The decision curve analysis of the nodule prediction model for postoperative sarcopenia in cases of patellar fractures is shown in Fig. 3C. This implies that the current prediction model can better guide clinical practice and facilitate early planning and implementation of clinical intervention to reduce the risk of sarcopenia, thus reducing the overall risk of muscle loss in the population.

**Table 1:** Differences between demographic and clinical characteristics of sarcopenia and normal group.

Characteristics			
	Sarcopenia (*n* = 45)	Normal (*n* = 92)	Total (*n* = 137)
**Gender**			
Female	29(64.4%)	56(60.9%)	85 (62.0%)
Male	16(35.6%)	36(39.1%)	52 (38.0%)
**Age**			
Mean (SD)	69.3(9.7)	63.9(7.3)	65.7 (7.8)
Median [MIN, MAX]	67 [55,88]	62[55,90]	63[55,89]
**Education_level**			
Primary (0–6 years)	10(22.2%)	16(17.4%)	26(19.0%)
Secondary (7–12 years)	24(53.3%)	49(53.3%)	73(53.3%)
Higher (>12 years)	11(24.4%)	27(29.3%)	38(27.7%)
**BMI**			
Mean (SD)	22.1(2.8)	24.7(3.2)	23.9(3.5)
Median [MIN, MAX]	22.8[17.5,26.8]	25[17.4,31.8]	24.2[17.4,31.8]
**Drinking**			
Yes	9(20.0%)	38(41.3%)	90(65.7%)
No	36(80.0%)	54(58.7%)	47(34.3%)
**Smoking**			
Yes	15(33.3%)	23(25.0%)	99(72.3%)
No	30(66.7%)	69(75.0%)	38(27.7%)
**Exercise**			
>2 h/week	24(53.3%)	75(81.5%)	99(72.3%)
≤2 h/week	21(46.7%)	17(18.5%)	38(27.7%)
**Surgical technique**			
Cannulated screws+Standard tension band	39(86.7%)	84(91.3%)	123(89.8%)
Standard tension band	4(8.9%)	7(7.6%)	11(8.0%)
Plaster fixation	2(4.4%)	1(1.0%)	3(2.2%)
**Rehabilitation training**			
Yes	13(28.9%)	47(51.1%)	60(43.8%)
No	32(72.1%)	45(48.9%)	77(56.2%)
**Internal fixation removal**			
Yes	13(28.9%)	37(40.2%)	50(36.5%)
No	32(72.1%)	55(60.8%)	87(63.5%)
**Diabetes**			
Yes	23(51.1%)	24(26.1%)	47(34.3%)
No	22(48.9%)	68(73.9%)	90(65.7%)
**High blood pressure**			
Yes	15(33.3%)	22(23.9%)	37(27.0%)
No	30(66.7%)	70(76.1%)	100(73.0%)
**Hyperlipidemia**			
Yes	12(26.7%)	25(27.2%)	37(27.0%)
No	33(73.3%)	67(72.8%)	100(73.0%)
**History of stroke or myocardial infarction**			
Yes	4(8.9%)	3(3.3%)	7(5.1%)
No	41(91.1%)	89(96.7%)	130(94.9%)
**Heart disease**			
Yes	6(13.3%)	8(8.7%)	123(89.8%)
No	39(86.7%)	84(91.3%)	14(10.2%)
**Renal insufficiency**			
Yes	1 (2.2%)	0(0.0%)	1(0.7%)
No	44 (97.8%)	92(100.0%)	136(99.3%)
**History of cancer**			
Yes	1 (2.2%)	2(2.2%)	3(2.2%)
No	44 (97.8%)	90(97.8%)	134(97.8%)
**Asthma**			
Yes	2(4.4%)	3(3.3%)	5(3.6%)
No	43(95.6%)	89(96.7%)	132(96.4%)
**Blood type**			
A	12(26.7%)	31(33.7%)	43(31.4%)
B	4(8.9%)	29(31.5%)	33(24.1%)
AB	15(33.3%)	8(8.7%)	23(16.8%)
O	14(31.1%)	24(26.1%)	38(27.7%)
**Perioperative blood transfusion**			
Yes	0(0.0%)	0(0.0%)	0(0.0%)
No	45(100.0%)	92(100.0%)	137(100.0%)
**Follow-up time(year)**			
Mean (SD)	3.7(1.6)	3.7(1.7)	3.7(1.7)
Median [MIN, MAX]	3.6[0.83,6.25]	3.46[8.3,6.25]	3.7[0.83,6.25]
**Length_of_hospital_stay**			
Mean (SD)	10.7(5.1)	10.3(4.5)	10.4(4.8)
Median [MIN, MAX]	10[3,24]	10[3,26]	10[3,26]
**Treatment group**			
Group A	19(42.2%)	42(45.7%)	61(44.5%)
Group B	26(57.8%)	50(54.3%)	76(55.4%)
**Bone nonunion**			
Yes	2(4.4%)	1(1.0%)	3(2.2%)
No	43(95.6%)	91(98.9%)	134(97.8%)
**Open fracture**			
Yes	5(11.1%)	3(3.3%)	8(5.8%)
No	40(88.9%)	89(96.7%)	129(94.1%)
**Multiple fracture**			
Yes	2(4.4%)	4(4.3%)	6(4.3%)
No	43(95.6%)	88(95.7%)	131(95.6%)
**Infection**			
Yes	4(8.9%)	3(3.3%)	7(5.1%)
No	41(91.1%)	89(96.7%)	130(94.9%)
**Classification of fracture**			
Upper or lower pole	17(37.8%)	32(34.8%)	49(35.8%)
Comminuted fracture	11(24.4%)	13(14.1%)	24(17.5%)
Transverse	15(33.3%)	38(41.3%)	53(38.7%)
Undisplaced	0(0.0%)	5 (5.4% )	5(3.6%)
Vertical fracture	2(4.4%)	4(4.3%)	6(4.3%)

##  Discussion

With the development and availability of R software, the nomogram prediction model is now being widely used in prognostic evaluation and assessment of treatment efficacy (Wang et al., 2019; Polterauer et al., 2012; Zhu et al., 2013). Additionally, the nomogram prediction model has well-defined quantitative indicators, which facilities accurate evaluation of treatment outcome and prognosis (Wei et al., 2017). In this paper, we describe, for the first time, the use of a nomogram model for the assessment of the risk of post-fracture sarcopenia.

In this study, we developed and validated a new tool for predicting postoperative sarcopenia in patients with patellar fractures by using only eight easily available variables. Our preliminary tests reveal that this model can serve as a relatively accurate predictor of whether or not muscle loss occurs in patients who undergo surgery for patellar fractures. Internal sampling showed that the model has strong predictive ability, while high values of C-index and AUC suggest that this model can be widely used for the prediction of postoperative sarcopenia in patients, with good accuracy (Wei et al., 2017).

In our study population, 31% of the patients developed sarcopenia. Analysis of the risk factors for sarcopenia included the following factors: age, BMI, diabetes, postoperative rehabilitation, frequency of outdoor exercise, surgical method used, presence or absence of open fractures, and removal of internal fixation. Our predictive model showed that low BMI, diabetes, lack of rehabilitation after surgery, lack of regular exercise and different surgical methods may be the key individual factors that lead to postoperative muscle loss.

As shown in several other studies, a decrease in BMI is often indicative of a decrease in the muscle strength of the limbs (Perna et al., 2015; Rondanelli et al., 2017), and patients with sarcopenia generally have relative low BMI (Perez-Sousa et al., 2019). Therefore, a decline in BMI is an important manifestation of sarcopenia in patients undergoing surgery after fracture. In addition, studies have also shown that very low weight is closely related to the occurrence of sarcopenia (Yu et al., 2014). For the elderly, while obese body types can induce and worsen many diseases, low body weight is also associated with poor health and is an important risk factor for death (Goh & Tong, 2010). High BMI can be considered as protective against the development of sarcopenia in the elderly (Yu et al., 2014). Therefore, maintaining a high BMI through intake of adequate nutrients can prevent the occurrence of postoperative sarcopenia in elderly fracture patients (Veronese et al., 2019).

More than 422 million people worldwide have been estimated to have diabetes (Cameron et al., 2008), and sarcopenia has been reported as a complication of diabetes (Kalyani et al., 2015; Trierweiler et al., 2018), Our study confirmed that diabetes was an important risk factor for muscle reduction in patients with patellar fractures. The incidence of sarcopenia has also been reported to be significantly high in patients with poor glycemic control, and sarcopenia in turn leads to the worsening of diabetes (Park et al., 2006). This is because in the presence of type 2 diabetes, insulin resistance results in autophagy, degradation of muscle protein (ubiquitin–proteasome proteolytic pathway), and mitochondrial dysfunction; these processes eventually lead to the loss of muscle mass and muscle strength. Muscle mass and loss of muscle strength, in turn, lead to a potential increase in insulin resistance; additionally, further progression of mitochondrial dysfunction can aggravate insulin resistance, ultimately compromising diabetes control and then stimulating pathways leading to further muscle loss (Fujita et al., 2009; Jerusalem, Engel & Peterson, 1975; Kalyani et al., 2012; Kaushik, Singh & Cuervo, 2010; Kelley et al., 2002; Mogensen et al., 2007; Phielix & Mensink, 2008; Wang et al., 2006). This vicious circle between muscle reduction and diabetes can be broken by rehabilitation exercise, which is known to have a beneficial effect on bone metabolism (Gao et al., 2016). Further, studies have shown that aerobic exercise with appropriate resistance training is an economic and effective intervention and that it is helpful for effectively controlling diabetes and improving the condition of elderly diabetic patients (Tan, Li & Wang, 2012). These findings are consistent with the results of our study, where our predictive model demonstrated that postoperative rehabilitation exercise and regular physical exercise can prevent the development of sarcopenia (Conceição & Ugrinowitsch, 2019).

The maximum load that the quadriceps can bear can reach 3200N, and the sputum can reach 2800N. In healthy young people, the load on the knee joint can reach 6000N or above (Huberti et al., 1984). This value refers to the load requirement to be achieved after surgery; strong and stable internal fixation is particularly important for postoperative rehabilitation of patellar fractures. The usage of cannulated screws with standard tension band can often help achieve stable internal fixation (Rafael & Archdeacon, 2016). In addition, conservative treatment (such as plaster splint) can be used for fractures with partial knee extension devices intact while long-term immobilization can lead to the development and deterioration of sarcopenia (Cohen, Nathan & Goldberg, 2015).

Interestingly, we found that implant removal is a predictor of functional recovery of the knee joint after surgery. Retention of internal fixation has many risks, including screw fracture, postoperative fracture, important tissue damage, major bleeding, intraoperative fracture, infection, poor wound healing, etc (Arora et al., 2007). However, with thorough preoperative preparation, these complications were avoided in our patients. Additionally, some patients may develop limited joint mobility due to the retention of internal fixation. Retention of internal fixation also hinders the removal of heterotopic bone and soft tissue release, so it must be taken out for complete release of the tissues (Park et al., 2010; Ring & Jupiter, 2004; Wang et al., 2014).

Our prediction model also highlights age as an important risk factors for the development of sarcopenia. Baumgartner et al. (1998) have shown that the incidence of sarcopenia in patients aged between 65 and 70 years ranges at 13–24%. Further, studies have also shown that sarcopenia may occur in as many as half of elderly subjects aged ≥80 years (Volpato et al., 2014; Von Haehling, Morley & Anker, 2010). The study also suggests that open fractures may be a poor prognostic factor for fracture patients; this is consistent with the findings of previous studies that have shown that open fractures led to more complications and prolonged hospitalization (Rees et al., 2019). Moreover, studies have also shown that prolonged hospitalization affects patients’ daily activities (Wanigatunga et al., 2019).

The predictive tool developed by us offers several benefits: (1) Using a predictive tool for postoperative muscle reduction in patients with patellar fractures enables individualized risk prediction, which can indirectly improve the overall patient outcomes (Kardas, Lewek & Matyjaszczyk, 2013). (2) Our model can serve as an effective sarcopenia risk prediction tool, which would enable clinicians to identify patients at a high risk of developing sarcopenia and take appropriate measures to prevent the same. (3) Further, our assessment tool can provide a theoretical background to guide further clinical research on sarcopenia. For example, our nomogram prediction model can be used to facilitate the selection of patients with high risk of sarcopenia in clinical trials. (4) Furthermore, simple early interventions, such as the engagement of medical reminders and family support, will help reduce the risk of postoperative sarcopenia in high-risk patients (Martone et al., 2017). In summary, patients undergoing post-fracture surgery should be recommended treatment for diabetes, post-operative rehabilitation, regular physical activity, and education regarding postoperative management in order to reduce the risk of sarcopenia (Abdelhafiz & Sinclair, 2015).

Therefore, an accurate prognostic assessment tool would enable physicians to evaluate the risk of sarcopenia in a timely manner and accordingly make necessary interventions to improve the prognosis of patients, while also eliminating the wasteful use of medical resources caused by false-positive results. Although it is difficult to completely eliminate the occurrence of sarcopenia, it may be certainly possible to reduce its risk through reasonable evaluation and cost-effective interventions. This would substantially improve the outcomes in patients undergoing post-fracture surgery and therefore our findings have important clinical significance.

Our current study also has a few limitations. First, our sample population contains fewer males than females due to the higher risk of fractures in the latter (Johansson, Nordstrom & Nordstrom, 2016). Thus, this cohort is not representative of all patients with patellar fractures. Second, for the risk factor analysis in this study, we did not include all the risk factors for muscle reduction after patellar fractures. Third, although our prediction model has good internal sampling, the inclusion of stable external data is still necessary for further validation. Fourth, the data used in this study were collected from the patients’ clinical history and long-term follow-up data; although we take all measures to ensure accuracy of the data as much as possible, because of the high patient number, it may not be entirely possible to rule out missing or incorrect information. Fifth, because of the limited sample size, surgical methods are limited.

In this paper, we described a new predictive model that helps clinicians assess the risk of sarcopenia with good accuracy, in elderly patients with patellar fractures. Our findings indicated that the most common risk factors for muscle reduction in patients after fractures include advanced age, low BMI, diabetes, and open fracture. On the other hand, postoperative rehabilitation training, regular outdoor exercise, removal of internal fixation, and strong internal fixation technique were identified as factors protecting against muscle loss. We believe that after further validation through statistical testing and trials on random population samples, this model can be economically and effectively used for predicting the of postoperative muscle reduction in elderly patients with patellar fractures. Accurate estimation of individual risks can help clinicians and patients adopt appropriate modifications in lifestyle and medical interventions. Further research is warranted to determine whether change in interventions based on this nomogram prediction model can effectively reduce the risk of sarcopenia and improve patient outcomes.

##  Supplemental Information

10.7717/peerj.8793/supp-1 Supplemental Information 1 Raw data

10.7717/peerj.8793/supp-2 Supplemental Information 2 Questionnaire

## Additional Information and Declarations

### Data Availability

The following information was supplied regarding data availability: Raw data is available as a Supplementary File.
