# Supplementary material for: Predicting the risk of sarcopenia in elderly patients with patellar fracture: development and assessment of a new predictive nomogram
Source: PeerJ. 2020 Apr 15;8:e8793. doi: 10.7717/peerj.8793 (PMC7166043; doi:10.7717/peerj.8793)
Supplement: Supplemental Information 2 [file peerj-08-8793-s002.doc]

您好！

       本问卷是常规的术后随访及临床调研项目，旨在了解骨折后身体机能康复等方面的状况，以便让患者了解自己的健康状况，并给予患者以合理的建议，及为临床统计提供数据。您的答案无所谓对错。本问卷填答的资料**绝对保密**，所以请您根据自己的真实情况填答，非常感谢您的合作！

Q1 编号 姓名

Q2 您的性别 1.男 2.女

Q3 您的年龄 岁

Q4 您的体重 kg

Q5 您的身高_________cm

Q6 您骨折前是否吸烟？ 1.吸烟 2.不吸烟

Q7 您骨折前是否喝酒？ 1.喝酒 2.不喝酒

Q8 请问你的受教育程度是___________

1. 初等 (0–6年) 2.中等（7-12年） 3.高等（大于12年）

Q9 请问您骨折术后是否发生骨不连？ 1. 是 2.否 3.不清楚

Q10 请问您骨折术后是否进行了康复训练？

1. 进行了康复训练 2.没有进行康复训练

Q11 您骨折前是否患有高血压？ 1.有高血压 2.无高血压

Q12 您骨折前是否患有糖尿病？ 1.有糖尿病 2.无糖尿病

Q13 您骨折前是否患有高脂血症？ 1.有高脂血症 2.无高脂血症

Q14 您术后半年后平均每周进行有氧运动的时间大于2小时吗？

1. 大于2小时 2.小于2小时

Q15 您是否已行内固定取出术？ 1.内固定已取出 2.内固定未取出

Q16 您骨折前是否曾患有以下疾病？(可多选)

1. 卒中史 2.心梗史 3.恶性肿瘤病史 4.心脏病史 5.哮喘史

Q17 您骨折前是否有输血史？ 1.有输血史 2.无输血史

Q1 No. Name

Q2 Gender 1. Male 2. female

Q3 ages years

Q4 weight kg

Q5 height cm

Q6 Did you smoke before fracture? 1.Smoking 2.No Smoking

Q7 Did you drink alcohol before fracture? 1.Drink 2.No

Q8 Your education level ___________

1. Primary (0-6 years)2. Medium (7-12 years)3. Higher (greater than 12 years)

Q9 Did you suffer from bone ununion after fracture? 1.YES 2.No. 3. I don't know

Q10 Did you have any rehabilitation training after fracture? 1.YES 2. No rehabilitation training

Q11 Did you suffer from high blood pressure before fracture? 1. YES 2. No hypertension

Q12 Did you suffer from diabetes before fracture? 1. YES 2.No diabetes

Q13 Did you suffered from hyperlipidemia before fracture? 1.YES 2. No

Q14 Did you spend more than two hours of exercise a week?

1.more than 2 hours 2. less than 2 hours

Q15 Have you removed the Internal fixation? 1. Internal fixation removed 2. NO

Q16 Have you suffered from any of the following diseases prior to your fracture?

(You can choose more than one)

1. History of stroke 2. History of myocardial infarction 3. History of malignant tumor 4. History of heart disease 5. History of asthma

Q17 Did you have a history of blood transfusion? 1. YES 2. No
